# Egyptian clinical practice guideline for kidney transplantation

**DOI:** 10.1080/2090598X.2020.1868657

**Published:** 2021-01-03

**Authors:** Ahmed A. Shokeir, Saddam Hassan, Tamer Shehab, Wesam Ismail, Ismail R. Saad, Abdelbasset A. Badawy, Wael Sameh, Hisham M. Hammouda, Ahmed G. Elbaz, Ayman A. Ali, Rashad Barsoum

**Affiliations:** a Urology and Nephrology Center, Faculty of Medicine, Mansoura University, Mansoura, Egypt; b Faculty of Medicine, Banha University, Banha, Egypt; c Nephrology Department, Al-Sahel Teaching Hospital, Cairo, Egypt; d Faculty of Medicine, Beni-Suef University, Beni Suef, Egypt; e Urology Department, Kasr El-Einy Medical School, Cairo University, Cairo, Egypt; f Faculty of Medicine, Sohag University, Sohag, Egypt; g Faculty of Medicine, Alexandria University, Alexandria, Egypt; h Faculty of Medicine, Assuit University, Assuit, Egypt; i Urology Department, Theodor Bilharz Research Institute, El Warraq, Giza, Egypt; j Nephrology Department, Kasr El-Einy Medical School, Cairo University, Cairo, Egypt

**Keywords:** Clinical practice guideline, kidney transplantation

## Abstract

**Objective**: To present the first Egyptian clinical practice guideline for kidney transplantation (KT).

**Methods**: A panel of multidisciplinary subspecialties related to KT prepared this document. The sources of information included updates of six international guidelines, and review of several relevant international and Egyptian publications. All statements were graded according to the strength of clinical practice recommendation and the level of evidence. All recommendations were discussed by the panel members who represented most of the licensed Egyptian centres practicing KT.

**Results**: Recommendations were given on preparation, surgical techniques and surgical complications of both donors and recipients. A special emphasis was made on the recipient’s journey with immunosuppression. It starts with setting the scene by covering the donor and recipient evaluations, medicolegal requirements, recipient’s protective vaccines, and risk assessment. It spans desensitisation and induction strategies to surgical approach and potential complications, options of maintenance immunosuppression, updated treatment of acute rejection and chemoprophylactic protocols. It ends with monitoring for potential complications of the recipient’s suppressed immunity and the short- and long-term complications of immunosuppressive drugs. It highlights the importance of individualisation of immunosuppression strategies consistent with pre-KT risk assessment. It emphasises the all-important role of anti-human leucocyte antigen antibodies, particularly the donor-specific antibodies (DSAs), in acute and chronic rejection, and eventual graft and patient survival. It addresses the place of DSAs across the recipient’s journey with his/her gift of life.

**Conclusion**: This guideline introduces the first proposed standard of good clinical practice in the field of KT in Egypt.

**Abbreviations**: Ab: antibody; ABMR: Ab-mediated rejection; ABO: ABO blood groups; BKV: BK polyomavirus; BMI: body mass index; BTS: British Transplantation Society; CAN: chronic allograft nephropathy; CDC: complement-dependent cytotoxicity; CKD: chronic kidney disease; CMV: cytomegalovirus; CNI: calcineurin inhibitor; CPRA: Calculated Panel Reactive Antibodies; (dn)DSA: (*de novo*) donor-specific antibodies; ECG: electrocardiogram; ESWL: extracorporeal shockwave lithotripsy; FCM: flow cytometry; GBM: glomerular basement membrane; GN: glomerulonephritis; HIV: human immunodeficiency virus; HLA: human leucocyte antigen; HPV: human papilloma virus; IL2-RA: interleukin-2 receptor antagonist; IVIg: intravenous immunoglobulin; KT(C)(R): kidney transplantation/transplant (candidate) (recipient); (L)(O)LDN: (laparoscopic) (open) live-donor nephrectomy; MBD: metabolic bone disease; MCS: Mean channel shift (in FCM-XM); MFI: mean fluorescence intensity; MMF: mycophenolate mofetil; mTOR(i): mammalian target of rapamycin (inhibitor); NG: ‘not graded’; PAP: Papanicolaou smear; PCN: percutaneous nephrostomy; PCNL: percutaneous nephrolithotomy; PKTU: post-KT urolithiasis; PLEX: plasma exchange; PRA: panel reactive antibodies; PSI: proliferation signal inhibitor; PTA: percutaneous transluminal angioplasty; RAS: renal artery stenosis; RAT: renal artery thrombosis;:rATG: rabbit anti-thymocyte globulin; RCT: randomised controlled trial; RIS: Relative MFI Score; RVT: renal vein thrombosis; TB: tuberculosis; TCMR: T-cell-mediated rejection; URS: ureterorenoscopy; (CD)US: (colour Doppler) ultrasonography; VCUG: voiding cystourethrogram; XM: cross match; ZN: Ziehl–Neelsen stain

## Introduction

This document integrates recent international guidelines with Egyptian experience and environmental and socioeconomic circumstances. The latter are dominated by a rich bioecological environment, specific demographics and social constraints, limited financial resources and exclusively live-donor transplantation due to inability to perform deceased donor kidney transplantation (KT) in Egypt at the current time. It is focussed on the adult recipient. While we touch on general issues related to children, pregnant women and elderly patients, we have kept this information brief, and cited selected comprehensive position statements that provide more details on these special populations.

## Methodology

Our recommendations are the outcome of integrating five resource categories:

- Six guidelines (including their latest updates), namely Kidney Disease Improving Global Outcomes (KDIGO), European Association of Urology (EAU), European Renal Best Practice (ERBP), British Transplantation Society (BTS), National Institute of Health Research (NIHR), and Australian Transplantation guidelines [1–6].
- Review of several guides, position statements, meta-analyses and leading institutional protocols [7–13].
- Relevant Egyptian publications.
- Results of a specifically designed 17-question online survey (using the SurveyMonkey^@^ software) to which the current and past leading nephrologists of Egyptian Ministry of Health-licensed transplant centres were recruited. A total of 40 nephrologists were invited, of whom 28 responded.
- A panel of six transplant surgeons representing most centres in Egypt practicing live-donor KT.

All statements were graded according to two parameters:

- *Strength of clinical practice recommendation*, expressed as a number from 1 to 3, 1 being highest. This reflects the consensus of the authors, survey responders, and an invited panel of 26 experts of nephrology and urology, and the Guidelines Committee of the Egyptian Society of Nephrology and Transplantation. While this was guided by other guideline recommendations, it was modified to suit the Egyptian environment dominated by a rich bioecological environment, specific demographics and social constraints, limited financial resources, and exclusivity of live-donor KT.
- *Level of evidence*, expressed as a letter, from A to D, based on scientific merit as valuated in international guidelines.

The relevant text was adapted to match the clinical recommendations as explained in Table 1.

**Table 1.** Evidence and clinical recommendations grading

Level of evidence		Strength of clinical recommendation		Strength rating in the Egyptian Guideline
A	High-quality RCTs with specific relevance	1	Strongly recommended	Strong
B	Moderate-quality RCT, broad relevance, meta-analysis	2	Recommended	Moderate
C	Registry data, patient cohorts, case/control studies	3	Suggested	Weak
D	Case reports, narratives, expert opinion	NG	Suggested	Not graded

‘Not graded (NG)’ means a lack of documented evidence yet based on experts’ experience or logical opinion. This may turn out to be clinically stronger than low-grade evidence.

All recommendations related to immunosuppression were discussed by two panels of experts, including the Cairo Kidney Center Transplant Team and the Egyptian Society of Nephrology Transplant Guidelines workgroup. Recommendations related to preparation, surgical approach, and surgical complications of donors and recipients were discussed by a panel of six urological surgeons.

## Guidelines

### Box 1: Living-donor evaluation

- We strongly recommend taking a detailed history including age, gender, body mass index (BMI), marital status, and consanguinity with the recipient (1B).
- We strongly recommend social and psychological assessment, and illicit drug testing. (1 C).
- We strongly recommend routine urine analysis and culture (1 C).
- We strongly recommend 24-h urine collection for protein and creatinine (1 C).
- We recommend Ziehl–Neelsen stain (ZN) and PCR for tuberculosis (TB) in urine for suspected cases (2 C).
- We strongly recommend routine blood tests including full and differential blood counts, renal function tests, liver function tests, blood electrolytes, lipid profile, viral profile testing for hepatitis B, hepatitis C, cytomegalovirus (CMV), and human immunodeficiency virus (HIV) (1 C).
- We strongly recommend radiological investigations in terms of as grey-scale ultrasonography (US), plain urinary tract X-ray, chest X-ray, CT angiography, CT urography, and radioisotope renography (1 C).
- We strongly recommend an electrocardiogram (ECG) (1 C).

### Box 2: Preoperative recipient evaluation

- We strongly recommend taking a detailed history including age, gender, BMI, marital status, duration of dialysis, original kidney disease and amount of urine output per day, history of previous surgery, and history of medications (1B).
- We strongly recommend routine urine analysis and culture (1B).
- We suggest ZN and PCR for TB in urine for suspected cases (3 C).
- We strongly recommend routine blood tests including full and differential blood counts, renal function tests, liver function tests, blood electrolytes, lipid profile, viral profile testing for hepatitis B, hepatitis C, CMV and HIV (1B).
- We suggest examination of sputum for TB by ZN and PCR for suspected cases (3 C).
- We recommend radiological profile including abdominal US, abdominopelvic CT, chest X-ray and micturition cystourethrogram (in cases with suspected voiding dysfunction or VUR) (2B).
- We recommend ECG, echocardiography and vascular assessment including vessels of the neck, pelvis and lower limbs as appropriate (2B).
- We suggest full urodynamic studies and urethrocystoscopy as appropriate (3 C).
- We suggest upper and/or lower gastrointestinal tract endoscopy as appropriate (3 C).
- We suggest biopsy from kidneys, liver, rectum as appropriate (3 C).
- We recommend exclusion of malignant diseases by thyroid evaluation, mammography in females, Papanicolaou smear (PAP) smear in females, and DRE and PSA in males (2B).

### Box 3: Medico-legal issues

- It is compulsory and mandated by the Egyptian Code for Organ and Tissue Transplantation that hospitals seeking to perform organ transplantation to be licensed by the Egyptian Ministry of Health.
- It is compulsory to obtain a fully informed consent from the donors and recipients.
- It is compulsory to obtain formal approval from the Egyptian Supreme Committee for Organ Transplantation for every case of KT.

### BOX 4: Vaccination

#### Kidney transplant candidate (KTC) (Pre-transplant)

- We strongly recommend all kidney transplant candidates to receive the inactive immunisation vaccines and boosters prior to transplantation for hepatitis B, *H. Influenzae*, seasonal influenza, meningococcus and pneumococcus (1 C).
- We suggest assessing immunogenicity by measuring available serum antibody (Ab) titres rather than previous history of infection or vaccination (3 C).
- We suggest giving live vaccines (measles, mumps, and rubella [MMR], herpes and varicella) if scheduled or National Health alerts and recommendations are issued, and to be at least 6 weeks prior to KT (2 C).
- We strongly recommend quadrivalent meningococcal conjugate vaccine for renal transplant candidates with previous splenectomy or planned to receive complement inhibitors (1 C).
- We suggest human papilloma virus (HPV) vaccine to be given to both sexes within the age range of 9 to 26 years (3 C).

#### Kidney transplant Recipient (KTR) (post-transplant)

- We recommend KT recipients (KTRs) to start or resume immunisation vaccines and boosters 6 months after KT except for seasonal flu (2 C).
- We strongly recommend seasonal flu vaccine for KTRs to be not earlier than 1 month after KT and before the onset of influenza season then annually (1 C).
- We strongly recommend avoiding all live vaccines for KTRs (1D).

#### Family and caregivers

- We suggest considering family and caregivers immunisation for enhancing KTRs protection (3 C).

The risk of community-acquired infection is significantly increased in KTRs, in parallel with the extent of immunosuppression. The incidence of pneumonia and influenza is multiplied many fold in KTRs compared to that in the general population. In the typical subtropical environment, including Egypt, KTRs are exposed to many other infections such as hepatitis, TB, typhoid, and others.

It is important to ensure adequate immune protection of KTRs by prior immunisation in accordance with the general population schedules. In case of historic uncertainty, primary or booster vaccination is recommended. In some vaccines, e.g. *H. Influenzae*, HBV, *S*. Typhi, etc., the level of protection can be measured by specific Ab levels, which determines the need for booster doses. Pre-transplant vaccination with live attenuated vaccines should be completed 4 weeks before KT to avoid activation upon immunosuppression, or 2 weeks with inactivated vaccines to provide enough time for acquiring adequate immunity. Vaccination is of confirmed value in reducing KTRs’ susceptibility to pneumococcal pneumonia, *H. influenzae*, seasonal influenza, hepatitis B new viral infection and meningococcal meningitis. The benefit of other vaccinations (Table 2 [14–16]) is less rigorously evidence based. Concerns about boosting transplant immunity by certain vaccines such as *H. influenzae* have been dismissed by adequate studies.

**Table 2.** Main recipient’s vaccination recommendations

	Pre-KT		Post-KT		
Vaccine	Survey responders, %	Recommendation	Survey responders, %	Recommendation	Comment
Inactivated Vaccines					
Diphtheria/Tetanus/Pertussis	25.9	+		+	
Haemophilus influenza B	44.4	+		+	Immunogenicity determined by Ab titre
Hepatitis B virus (HBV)	85.2	+		+	Target HBs Ab titre >10 IU/mL [14]
HPV	25.9	+		+	Quadrivalent vaccine aged <26 years [15]
Meningococcal	14.2	+		+	Quadrivalent conjugate
Pneumococcal	63.0	+	61.6	+	PCV13 followed by PPV23 ≥ 8 weeks apart. PPV23 booster annually
Polio		+		+	6 months post-KT
Salmonella Typhi (inactivated)		+		+	
Seasonal inactivated influenza	51.9	+	84.6	+	Trivalent inactivated formulation containing two A strains and one B strain [16]
Live attenuated vaccines					
Herpes Simplex (HSV)		+		±	
MMR		+		_	
Salmonella Typhi (attenuated)		+		±	Use only inactivated vaccine if necessary post-KT
Varicella-Zoster (VZV)	22.2	+		_	Use recombinant vaccine if necessary post-KT
Yellow fever		+		_	Risk of encephalitis post-KT
BCG		_		_	Contraindicated pre- and post-KT
Small Box		_		_	Contraindicated pre- and post-KT

(+) = recommended, (–) = not recommended, (±) = recommended if necessary post-KT.

Maintaining adequate infection-specific immunity *after* KT should be ensured by measuring specific Ab levels or following public health booster doses starting >1 month after KT. But owing to the supervening immunosuppression, live attenuated vaccines must be avoided, both in the recipient and his/her contacts.

It is crucially important that healthcare workers and contacts of transplanted recipients to be fully immunised and particularly for influenza with an inactivated vaccine [17].

### BOX 5: Risk assessment

- We strongly recommend human leucocyte antigen (HLA) typing of KTCs and donors, using molecular methods preferably Ab titration methods to remove inhibition (1B). Typing should include at all loci (1D) including DQ (1D) and DP in sensitised recipients (2D).
- We strongly recommend testing for anti-HLA Abs in all KTRs by solid phase assay (1B).
- We strongly recommend a pre-transplant assessment of the recipient’s past and present immunological risk factors, including DSAs (1D).
- In the re-transplant population, we recommend a higher risk score for historic DSAs to repeat mismatch especially at class II compared to other recognised sources of sensitisation (1 C).
- We recommend using the recipient’s immunological risk for individualisation of immunosuppressive therapy and post-KT monitoring (2D).
- We suggest not routinely testing KTCs for non-HLA Abs (2 C).

There is evidence that the incidence of acute rejection and the eventual long-term graft outcomes are related to pre-transplant recipient’s immunological risk factors. Most of the relevant recent literature, as well as 89.3% of our survey respondents, recommend pre-transplant risk assessment including historic data, and detection of circulating anti-HLA Abs by complement-dependent cytotoxicity (CDC) and solid-phase techniques (Luminex®). Unfortunately, a quantitative tool for measuring the collective recipient’s immunological risk is not yet available. Pending the development of such a validated tool, the main players may be categorised into five classes (Table 3).

**Table 3.** Proposed categorisation of recipient’s immunological risk

CAT A	Highest risk – *KT is contraindicated*: Positive CDC-XM; Positive FCM-XM MCS >250; Positive DSA RIS ≥17
CAT B	Very High risk – *requires desensitisation* Positive FCM-XM MCS ≤250; Positive DSA RIS <17; Historic positive DSA
CAT C	High risk – *Possible desensitisation; induction mandatory; modified immunosuppression and follow-up protocol* Previous graft failure due to rejection during first post-KT year, re-transplant, full HLA mismatches; CPRA >80% positive non-DSA anti-HLA Abs
CAT D	Intermediate risk – *Induction, modified immunosuppression and follow-up* Re-transplant; >3/6 HLA mismatches, CPRA 20–80%, *positive non DSA anti-HLA Abs*
CAT E	Low risk – Lacking all of the above factors, proportionate to HLA matching and without anti HLA Abs

CDC: complement-dependent cytotoxicity; DSA: donor-specific Abs; FCM: flow-cytometric; MCS: mean channel shift; CPRA: Calculated Panel Reactive Antibodies; RIS: Relative Mean Fluorescence Intensity (MIF) Score (10 points for each MFI ≥10 000 + 5 points for each MFI 5000–9999 + 2 points for each MFI 2000–4999); XM: Cross-match.

### BOX 6: Desensitisation

- We recommend offering desensitisation to KTRs with ‘high’ to ‘very high’ immunological risk (Table 3).
- We strongly recommend offering desensitisation for KTRs with immunological barrier to their potential donors due to both/either ABOi (1B) or HLAi (1 C).
- We strongly recommend desensitisation in the context of direct kidney donation (Ab reduction) or Kidney Paired Donation program (Ab avoidance) (1 C).
- We suggest escalating individual desensitisation protocols (in terms of frequency and modality: intravenous immunoglobulin (IVIg) + plasma exchange (PLEX) + rituximab ± others) to achieve pre-defined acceptable CDC, flow cytometric and solid-phase parameters, taking individual centre experience and total cost into consideration (NG).
- We suggest for individual transplant centres to set their own acceptable immunological targets to be achieved by desensitisation before proceeding to KT (NG).
- We suggest to follow-up DSAs after KT in desensitised KTRs (NG).
- We suggest avoiding desensitisation and searching for a different donor in recipients who remain in the ‘highest risk’ category (Table 3) (NG).

Desensitisation is the logical clinician’s response to the increasing disclosure of preformed anti-HLA Abs in KTRs [18]. It has permitted successful transplantation against ABO as well as HLA-incompatibility barriers. Desensitisation against ABO-incompatible donors has achieved remarkable advances nearing the outcomes of ABO-compatible transplants. However, the outcomes of desensitisation against HLA incompatibility remain poor especially with high Class II DSAs with documented post-desensitisation Ab-mediated rejection (ABMR) of 25–50% [19]. There are no compelling thresholds for, and contraindications to the use of desensitisation; different transplant centres are required to set their own. Generally speaking, desensitisation is required with moderate mean fluorescence intensities (MFIs) of DSAs, usually >2000, with relative MFI score (RIS) <17, with a mean channel shift (MCS) <250 in flow cytometry (FCM)-cross match (XM) and a negative CDC-XM (Table 3). Desensitisation may also be used to reduce non-DSA anti-HLA Abs, which has a negative impact on graft outcomes. Our survey responses show that Egyptian centres have widely variable indications and thresholds (14.8–66.7% under different circumstances).

There are many protocols for desensitisation, which should be tailored to the magnitude of DSAs in terms of their number and MFIs. Most popular desensitisation protocols use either high-dose IVIg (2 g/kg), or PLEX (or immunoadsorption) + low dose IVIg (100 mg/kg after each session). Other protocols add, as yet off-label, rituximab, bortezomib, obinutuzumab, imlifidase, eculizumab, tocilizumab, or IgG endopeptidase, etc. DSAs must be measured again after desensitisation to ensure achievement of pre-set targets. Owing to the considerable inter-laboratory variability of current MFI measurements, each centre is required to set its own targets.

### Box 7: Donor nephrectomy

- We strongly recommend transplanting the kidney with the lesser function and/or minor anomaly (1 C).
- We recommend considering a right donor nephrectomy in female donors of the child-bearing age to avoid potential right hydronephrosis during pregnancy (2D).
- We strongly recommend that open live-donor nephrectomy (OLDN) be performed by experienced transplant urologists in centres where laparoscopic equipment and experts are not available (1A).
- We strongly recommend performing laparoscopic LDN (LLDN) in well-equipped highly specialized centres with trained staff only (1A).
- We suggest offering long-term annual monitoring of all living kidney donors by physical examination and measuring urinary protein excretion, serum creatinine and estimated GFR (eGFR) (3 C).

OLDN is still a valid approach in KT; however, the open approach is associated with an increased risk of wound complications, as well as poor cosmetic outcome. Although the warm ischaemia time is longer in LLDN when compared to OLDN, there is no difference in final graft function [20,21].

LLDN is as safe as OLDN regarding donor and graft survival, rejection, and urological complications in addition to the superior results in terms of shorter hospital stay and time to return to work, and less intraoperative blood loss [22]. The risk of end-stage renal disease and survival rate of kidney donors, are similar to those in general population with consistent donor satisfaction. However, we still need to develop a registry for the long-term follow-up [23].

### Box 8: Surgery on the renal transplant recipient

#### Surgical approach

- We strongly recommend that the KT surgery be performed by a well-trained team lead by a highly experienced transplant urologist (1A).
- We strongly recommend open KT as the standard surgical approach (1A).
- We suggest perivascular lymphatics to be carefully ligated (NG).
- We suggest meticulous mobilisation of appropriate segments of both the iliac artery and vein (NG).
- We suggest cooling the surface of the kidney with ice bags/slush during implantation (NG).

#### Venous anastomosis

- We strongly recommend that the renal vein (equally right or left) is anastomosed to the external iliac vein end-to-side (1B).
- In case of a short renal vein (usually right-sided donor kidney), we recommend tension-free anastomosis by ligating and dividing the recipient’s internal iliac vein (2B).

#### Arterial anastomosis

- We strongly recommend repairing any intimal rupture or flap in either donor or recipient arteries prior to anastomosis to avoid iliac artery dissection (1A).
- We recommend a single renal graft artery to be anastomosed to internal iliac artery (end-to-end) or to the external or common iliac artery (end-to-side) (1B).
- We suggest that two, equal size, graft arteries can be anastomosed separately or anastomosed together to create one ostium (3 C).
- We suggest that two, unequal size, graft arteries can be anastomosed separately (3 C).
- We suggest that anastomosis is achieved using 5/0 or 6/0 non-absorbable monofilament polypropylene sutures (3 C).
- We recommend that time of vascular anastomosis and hence graft re-perfusion time should be kept to minimum for better graft function (2B).

#### Ureteric anastomosis

- We strongly recommend that native ureter should be kept to an appropriate length to avoid kinking or redundancy in addition to preserving its periureteric fat to preserve its blood supply (1B).
- We strongly recommend that ureter is anastomosed to the bladder by an extravesical non-refluxing (Lich–Gregoir) uretero-neocystostomy (1A).
- We recommend stenting the uretero-neo-cystostomy (2A).
- We recommend removing the stent within 30 days (2 C).

The kidney (whether right or left) can be placed in either the right or left iliac fossa. An extraperitoneal iliac fossa ‘hockey-stick’ Gibson incision is the standard approach [24]. The fatty tissue surrounding the kidney should be dissected off the vessels. Branches that drain into the renal vein (adrenal or gonadal) should be ligated. Fatty tissue close to renal hilum should be ligated to reduce postoperative lymphocele formation. This preparation may be done before exploring the recipient. In the surgical bed, the perivascular lymphatics should be carefully ligated to minimise the development of postoperative lymphocele formation. The site of vascular anastomosis should be carefully identified, so as to avoid kinking [25].

Tension-free anastomosis of the donor renal vein is mandatory. Transposition of the recipient iliac artery and vein can help to achieve that. The renal vein can also be lengthened using either donor gonadal vein or recipient saphenous vein [26].

The internal iliac artery is more frequently affected by atherosclerosis than the external or common iliac arteries and so less preferred for end-to-end anastomosis with the donor artery. Multiple renal graft arteries should be handled carefully to ensure proper graft reperfusion. In case of two renal graft arteries and one is small it can be either sacrificed (if possible), anastomosed to the main renal artery (end-to-side), or anastomosed to the inferior epigastric artery (end-to-end). For multiple arteries, reject the donor or combine more than one of the previously mentioned techniques. Finishing the vascular anastomosis, unclamp the renal vein first then the artery [27].

In a normal bladder with no underlying abnormality, the well-vascularised graft ureter can be anastomosed to the bladder by either an extravesical (Lich–Gregoir) or intravesical (Politano–Leadbetter) uretero-neo-cystostomy using monofilament absorbable sutures with the former more preferred for its reduced overall complications and less UTIs. When the graft ureter is short, ischaemic, or denuded, the surgeon should use the native ureter for ureteroureterostomy or pyeloureterostomy if they are completely in a healthy condition. Although ureteric stenting is preferable as it may help to prevent major urological complications, it may be omitted to avoid another endoscopic procedure to be removed [28]. Duplex ureters can be anastomosed separately or combined following the same principles.

### Box 9: Recipient’s vascular complications

#### Haematoma and haemorrhage

- We suggest image-guided drainage or exploration for large post-KT haematomas causing graft pressure or haemodynamic instability (3 C).

#### Renal vein thrombosis (RVT)

- We recommend performing colour Doppler US (CDUS) if RVT is suspected (2B).
- We recommend surgical exploration if graft shows impaired perfusion on CDUS (2B).
- We recommend performing thrombectomy if RVT is confirmed with a viable graft. Similarly, we suggest performing a graft nephrectomy if RVT is confirmed with an unviable graft (2B).
- We strongly recommend not using routine prophylactic anticoagulation to prevent RVT in low-risk patients (1B).

*Renal artery thrombosis (RAT)*

- We recommend performing CDUS if RAT is suspected (2B).
- We recommend urgent surgical exploration if the graft shows impaired perfusion on CDUS (2B).
- We recommend performing thrombectomy if RAT is confirmed with a viable graft (2B).
- We recommend performing graft nephrectomy if RAT is confirmed with an unviable graft (2B).

#### Renal artery stenosis (RAS)

- We strongly recommend performing CDUS if RAS is suspected with MR or CT angiography performed for equivocal cases (1A).
- We suggest performing percutaneous transluminal angioplasty (PTA)/stenting as first-line for RAS (3 C).
- We suggest performing surgery for failed PTA, or long, multiple, or narrow RAS (3 C).

Haematomas occur frequently after KT (0.2–25%) being mostly minor and asymptomatic and requiring no intervention. Larger haematomas may be associated with graft dysfunction due pressure, or haemodynamic instability requiring image-guided drainage or exploration [29,30].

A RVT occurs early and rarely after KT (0.1–0.5%) [29–33]. It is responsible for up to 75% of early allograft loss [34]. Surgical errors, difficulties, and hyper-coagulative states are the main risk factors [33]. CDUS shows a swollen graft, reduced or no venous flow, and a plateau reversed diastolic arterial signal [35]. Immediate exploration is indicated. If the graft is salvageable (rare event) a venotomy with thrombectomy should be done with explantation, flushing, and re-implantation [30]. Thrombolytic agents and routine postoperative heparinisation were not useful as **prophylaxis** [36]**, but could be of help in treatment** [37].

A RAT is rare (1%) [29,31–33]. It results from poor technique, donor and recipient arterial intimal trauma and atherosclerosis, size disparity, kinking, hypotension, thrombophilia, acute rejection, external compression (haematoma or lymphocele), and immunosuppression toxicity. A RAT presents with acute oliguria and rising creatinine, and usually results in graft loss. CDUS is of choice if a RAT is suspected. It shows absence of flow in the main and intrarenal arteries [25]. Immediate surgical exploration is imperative for graft assessment. If the graft is salvageable, thrombectomy with graft flushing and revascularisation are done. In most cases, the graft is not salvageable, thus requiring nephrectomy. Selective angiographic thrombolytic therapy through the renal artery can be an alternative if a RAT is diagnosed <24 h and >10 days following KT [29,31–33]. Routine postoperative anticoagulation does not reduce rate of RAT in low-risk patients [36].

A RAS has a reported wide incidence range (1–25%) [29–32], due to different imaging protocols. It occurs from months to years after KT. A RAS may result in worsening graft survival [38]. Donor and recipient artery (size, arteriosclerosis and trauma), suturing (running or interrupted), recipient age, CMV, rejection, and delayed graft function are risk factors [38]. It presents with worsening and/or refractory hypertension and/or graft dysfunction without other reasons for graft dysfunction. On CDUS, a peak systolic velocity >200 cm/s in allograft artery is diagnostic. MR, CT, or a conventional angiogram can be performed [32,39]. A RAS can be haemodynamically insignificant or significant (< or >50%, with or without refractory hypertension, or with or without graft dysfunction). If insignificant, no intervention is needed (only CDUS surveillance). If significant, angiographic angioplasty/stenting, or surgery (recent KT, multiple, long and narrow stenosis, or failed angioplasty) are options [38].

### Box 10: Recommendations for the recipient’s urological complications

#### Urine leakage

- We suggest managing urine leak by stenting catheter and/or percutaneous nephrostomy (PCN) (3 C).
- We recommend performing surgery when conservative measures fail (2B).

#### Ureteric obstruction

- We recommend placing a PCN for the relief of obstruction and antegrade pyelogram for stricture delineation (2B).
- We suggest managing strictures <3 cm endoscopically (antegrade balloon dilatation or laser incision using flexible ureterorenoscopy [URS]) (3 C).
- We recommend managing recurring strictures or strictures >3 cm with surgery (2B).

#### Lymphocele

- We suggest performing aspiration or percutaneous drain placement as first-line in large and/or symptomatic lymphoceles with injection of sclerosing agents (3 C).
- We suggest performing laparoscopic or open marsupialisation if percutaneous drainage fails (3 C).

#### Urolithiasis

- We recommend attempting to identify the cause of post-KT urolithiasis (PKTU) (2B).
- We recommend placing a ureteric stent or PCN if PKTU is causing obstruction (with graft hydroureteronephrosis and/or impaired kidney function) (2B).
- We recommend performing ESWL or URS (ante- or retrograde) for stones <15 mm (2B).
- We recommend performing percutaneous nephrolithotomy (PCNL) for stones >20 mm (2B).

#### Vesico-ureteric reflux (VUR)

- We suggest performing endoscopic injection of bulking agent as the first-line for symptomatic or clinically relevant VUR (3 C).

Urinary leakage occurs in 0–9% [40,41]. Leaks can be from the ureter or bladder due to surgical error or ureteric necrosis. They usually occur within 2–4 weeks of KT and present with decreased urine output, abdominal pain and distention, fever, excessive drainage of fluid from the drain or incision, with a high creatinine level. Preserving periureteric fat during harvesting maintains ureteric blood supply. Routine stenting is controversial with reports recommending it [33]. A recent systematic review and meta-analysis concluded that routine stenting does not lower the incidence of urine leakage or urological complications, and recommended stenting only in pathological bladders [42]. To identify the leakage site US, CT, voiding cystourethrogram (VCUG), or antegrade pyelogram maybe done. Small and/or early leaks maybe managed with prolonged catheterisation, PCN, or stenting. Failed conservative management or massive leakage requires redo surgery (ureteroneocystostomy or use of native ureter) [33].

Ureteric stenosis occurs in 1–6% of KTs [33,43]. Early stenosis is due to ureteric ischaemia or poor technique. Preservation of lower polar arteries and periureteric fat help prevent ureteric ischaemia. Late stenosis maybe due to infection, rejection, BK polyomavirus (BKV), vascular disease, lymphocele, or stones [33]. US, CT, renography, VCUG, and retrograde or antegrade pyelogram are diagnostic. Intervention is needed with worsening hydroureteronephrosis or kidney function [33], while depending on timing, location and length of stricture, patient factors, and surgeon preference. Short strictures (<3 cm) maybe endoscopically managed with ante- or retrograde balloon dilatation or flexible URS with holmium laser incision. For strictures <1 cm endoscopic laser incision was superior to balloon dilatation (50% success) [44]. Recurrence after endoscopy or strictures >3 cm require surgical reconstruction (ureteroneocystostomy, pyelovesicostomy, or ureteroureterostomy using native ureter) [42].

Lymphoceles commonly occur after KT (1–26%). Risk factors are diabetes, rejection, delayed graft function and mammalian target of rapamycin inhibitors (mTORis) [40,45]. Lymphoceles maybe small and asymptomatic, diagnosed with routine imaging, and requiring observation. Others maybe large and symptomatic, warranting intervention. Image-guided aspiration can be performed albeit having a high recurrence rate (up to 95%) [46]. Percutaneous drain placement is considered first-line management achieving success in up to 50%. Injection of sclerosing agent (ethanol, tetracycline or fibrin glue) improves results. Laparoscopic and open surgical marsupialisation achieves the highest success and lowest recurrence rates [45–47].

PKTU occurs in 0.17–1.8%. PKTU can worsen graft function and survival due to obstruction and sepsis [40,46]. Risk factors include obstruction, reflux, recurrent UTIs, renal tubular acidosis, hyperfiltration, supersaturated urine, hypocitraturia, hypercalcaemia, hypercalciuria, hyperoxaluria, hyperuricaemia, and tertiary hyperparathyroidism [48]. Patients present with fever, increased creatinine, UTI, anuria, or a palpable mass due to hydronephrosis. Renal colic is usually not present due to graft denervation during harvesting. US is diagnostic; however, CT confirms stone size, location, number, and density [49]. Management depends on stone size, location, and obstruction. Stones with obstruction require initial PCN or stenting. Small stones (<1.5 cm) can be managed with ESWL (40–80% success). Antegrade or retrograde URS may be done for stones <2 cm (success close to 70%) [50] and PCNL for stones >2 cm [49].

The true incidence of VUR in KT is not clear as VCUG is not routine. When VCUG has been done to evaluate recurrent pyelonephritis, VUR was rare (0–2%). If the VCUG is routine, asymptomatic VUR may be diagnosed in up to 86% despite tunnelled uretero-neocystostomies. A dysfunctional LUT was linked to higher risk of VUR. UTI and CMV were associated with increased graft pyelonephritis. Recent reports did not link VUR to lower graft survival [41]. Low-grade VUR can be managed with antibiotic prophylaxis [51,52]. Symptomatic VUR can be managed with endoscopic injection of bulking agent (success in 80%) [41]. Re-do ureteroneocystostomy or ureteroureterostomy to native ureter are options [53].

### BOX 11: Induction of KTR immunosuppression

- We recommend including induction therapy with a biological agent as part of the initial immunosuppressive regimen in KTRs except Caucasians with 2-haplo-type identical living-related donor (2A).
- We recommend using an interleukin-2 receptor antagonist (IL2-RA), rather than no induction for KTRs at low immunological risk (2 C).
- We recommend using a lymphocyte-depleting agent, rather than an IL2-RA, for KTRs at high immunological risk (2B).

There is strong evidence that biological induction reduces the incidence of acute rejection and conserves better graft function. However, there is no evidence of improved graft or patient survival [6]. Only 29.6% of our survey responders used biological induction in all recipients, 59.2% in high-risk recipients only and 37.0% limited biological induction to high HLA mismatches (>50%). The available agents are IL2-RA, rabbit anti-thymocyte globulin (rATG), and Alemtuzumab. The latter is infrequently used owing to concerns about its long-term outcomes and increased frequency of autoimmune disease (e.g. anti-glomerular basement membrane [GBM] disease). An IL2-RA is a safe and effective agent suitable for low- and some intermediate-risk recipients, while the stronger rATG is recommended for high-risk patients, despite the associated risk of CMV activation and lymphoma.

Owing to the reduced immunological risk in patients aged >65 years, and their increased susceptibility to post-KT infection, many centres avoid biological induction without increased incidence of acute rejection. However, biological induction remains necessary if a steroid-free protocol is contemplated.

### BOX 12: Maintenance immunosuppression

- We strongly recommend starting a combination of immunosuppressive medications before, or at the time of, KT (1A).
- We strongly recommend initial triple immunosuppression with tacrolimus, mycophenolate and prednisolone in the high therapeutic range, regardless of the recipient’s immunological risk. (1A).
- If tacrolimus or mycophenolate cannot be used, we recommend replacement of tacrolimus with cyclosporine (2B) and suggest using azathioprine instead of mycophenolate, particularly in low-risk KTRs (3D).
- We strongly recommend frequent measurement of the blood levels of calcineurin inhibitors (CNIs) (1B) during the first 3 months for adequate dose adjustment, and less frequently thereafter (2 C) (associated with clinical follow-up visits), as well as with any acute deterioration of graft function, significant vomiting or diarrhoea, whenever drug-related side-effects are suspected, or with the introduction or withdrawal of drugs known to interact with CNI absorption or metabolism (NG).
- If an approved generic is used, we strongly recommend measuring the drug blood levels before and after switching and adjusting the doses accordingly (1A).
- If the therapeutic level of the CNI is difficult to achieve for clinical, pharmacokinetic or economic reasons, we suggest using a non-dihydropyridine calcium channel blocker or ketoconazole [54], with due attention to their side-effects, particularly the latter (NG).
- We suggest measuring mycophenolate trough blood level by the end of the first month post-KT to adjust the blood level at that recommended by the manufacturer, and as necessary thereafter whenever over- or under-immunosuppression is suspected. (3D).
- We recommend maintaining the higher limit of the CNIs and anti-proliferative drug blood level ranges for the first 3 months, then changing the target according to the recipient’s immunological risk category, aiming at the lowest therapeutic blood levels in low- or intermediate-risk KTRs if there has been no acute rejection (2 C).

Although all recent guidelines recommend starting immunosuppression upon hospital admission or at the time of KT, several live-donor KT protocols suggest starting maintenance immunosuppressive drugs 1–2 days prior to KT (92.8% of survey respondents). The rationale is to ensure the recipient’s achievement of a therapeutic blood level at the time of first exposure to the donor’s antigens (2 C).

The current standard prophylactic immunosuppression protocol (tacrolimus/mycophenolate mofetil/prednisolone [Tac/MMF/P]) is the outcome of high-quality evidence and extensive clinical implementation worldwide (1A). It is the preferred protocol by 88.9% of our survey’s respondents. Long-acting tacrolimus and mycophenolic acid are as safe and effective as short-acting tacrolimus and mycophenolate mofetil respectively, with certain pharmacokinetic or pharmacodynamic advantages.

There are acknowledged alternatives to standard Tac/MMF/P protocol, which are not inferior regarding graft survival at least at 1 year [5]. However, there are issues that make them less preferred. For example, the incidence of biopsy-confirmed acute rejection is higher with cyclosporin A/azathioprine/P (CSA/Aza/P) and with most *de novo* CNI-free protocols. There are concerns about the early use of proliferation signal inhibitors (PSIs) regarding the increased incidence of biopsy-confirmed acute rejection when used without CNIs, delayed wound healing, proteinuria, anaemia, pneumonia, etc.

CNIs have a narrow therapeutic window, hence the need for frequent blood level monitoring. Achievement of the target blood levels (Table 4) is of fundamental importance particularly during the first 3 months post-KT. Although generics are supposed to be pharmacologically identical to the patent drug, there may be some differences in their bioavailability, which necessitates checking blood levels and subsequent dose adjustment upon cross switching.

**Table 4.** Recommended therapeutic blood levels of the calcineurin inhibitors (CNIs)

CNI		First 3 months	4–6 months	6–12 months	After 1-year rejection free	Recommendation
Tacrolimus, ng/mL	C0	8–12	5–8	5–8	5–6	2D
Cyclosprine A, ng/mL	C0 C2	300–350 1300	150–250 800–900	100–150 500–700	75–125 450–500	2D

C0: trough level; C2: blood level 2 h after the dose

Mycophenolate, on the other hand, has a wider therapeutic window, has fewer drug–drug interactions, and its blood level is less volatile. Therefore, the need for monitoring is less pressing than with CNIs (2D).

The only exception to the 3-month immunosuppression stability rule is the steroid elimination protocol. The latter requires complete steroid withdrawal by 1 week post-KT in order to avoid subsequent acute rejection. If prednisolone is used beyond the first week, we suggest continuation for at least 1 year (2 C). Although these recommendations are adopted by most centres, there are other less popular protocols as shown in Table 5 [55].

**Table 5.** Steroid minimisation/elimination protocols

	Steroid-free maintenance regimes	Lower maintenance dosages	Complete avoidance	Early withdrawal	Late withdrawal
Strategy	Stoppage within 1 week post-KT	0.05–0.1 mg/kg by 1 year post-KT or sooner	None at induction or even at AR	Withdrawal within weeks to months	Withdrawal after years
Evidence level	1 USA prospective trial comparing stopping steroids vs continuation at low doses. Most other evidence from single centres	1 prospective trial comparing stopping steroids vs continuation at low doses. Most other evidence from single centres	Single centre studies without ethnic variation or immunologically high-risk patients, Mostly unverified from registry data	1 Canadian multicentre randomised double-blind clinical trial with two arms – stoppage at 90 days or continuation as alternate days	1 Meta-analysis 1 non-randomised European trial Other small studies
Adverse outcomes	Increase in CAN by ×2. Steroid side-effects same as in very low dose maintenance	CAN only at half the rate of very early withdrawal Steroid side-effects same as in very early withdrawal	1-year analysis in deceased-donor group 16% vs 11% in living donors	Significantly decreased long-term survival Adverse allograft survival from steroid withdrawal only evident at 5 years	34% excess risk of graft failure, 14% chance of AR Concurrent MMF use in late steroid withdrawal can be beneficial

AR: acute rejection; CAN: chronic allograft nephropathy.

### BOX 13: Modifications of maintenance immunosuppression

- In stable grafts, we strongly recommend that CNIs be continued, rather than withdrawn, unless clearly indicated (1B).
- We strongly recommend female KTRs who wish to become pregnant to be switched from mycophenolate to azathioprine at least 6 weeks before attempting pregnancy (1A).
- Female KTRs must switch mycophenolate to azathioprine once she discovers a confirmed pregnancy (NG).
- We suggest reducing CNI blood level targets in KTRs aged >65 years with stable grafts, taking the patient’s immunological risk grade into consideration (NG).
- If a steroid elimination protocol is contemplated, we recommend limiting its use to low-risk KTRs, using biological induction, and complete steroid withdrawal by 1 week post-KT (2 C) and to be considered for paediatric recipients.
- We recommend intensifying maintenance immunosuppression following recovery from an acute rejection, either by increasing the target drug blood levels, or by switching patients on cyclosporine to tacrolimus and/or azathioprine to MMF (2 C).
- In patients with rising serum creatinine attributed to the use of CNIs, we suggest switching to a PSI-based protocol (preferably everolimus [6] with either CNI minimisation or MMF [56], or to a Belatacept (in Epstein–Barr virus [EBV]-positive recipients), CNI-free protocol (NG).
- We recommend female KTRs to stop m-TOR inhibitors 3 months before attempting pregnancy, to be replaced as appropriate (2D).
- We recommend that male KTRs be advised that m-TOR inhibitors reduce the male sperm count and are counselled accordingly (1 C).

There is evidence that long-term standard triple immunosuppression is effective for an indefinite duration. Even though CNIs may induce interstitial fibrosis, this rarely progresses to graft failure despite a slowly rising serum creatinine. So, stable grafts may remain on this protocol indefinitely, which is the preferred policy in 67.9% of our survey respondents. As few as 10.7% and 7.1% preferred CNI withdrawal at 3 and 12 months respectively, while 14.3%, 7.1% and 14.3%% preferred steroid withdrawal at 3, 4, and 12 months post-KT, respectively.

Nevertheless, there are a few exceptions where minimisation or elimination of one or more of the triple immunosuppression components is required for a variety of reasons. The latter include age limitations, pregnancy, drug side-effects or idiosyncrasy, significant drug–drug interactions, infections or other post-KT complications.

The CARMEN study showed the benefit and safety of early steroid withdrawal by the first week post-KT. This concurs with an Egyptian study where steroids were administered for only 3 days [57].

Some centres prefer to switch even with stable grafts to a CNI-free protocol for the sake of better graft function [58]. Everolimus was shown to achieve this target, and also to prolong patient survival. Data on sirolimus are conflicting, both improved and reduced patient survival has been reported [6]. The m-TOR inhibitors should be avoided in pregnancy and in patients with urinary albumin/creatinine ratio >0.85. Their blood level should be regularly measured to achieve target.

CNI replacement with Belatacept has also been shown to improve graft function and histology and prolong patient survival [6]. However, Belatacept should not be used in EBV-negative recipients owing to the augmented risk of post-transplant lymphoproliferative disease.

### BOX 14: Immunosuppression in acute rejection

- We strongly recommend intravenous ‘pulse’ (‘bolus’) corticosteroids for the initial treatment of acute T-cell-mediated rejection (TCMR) followed by gradual tapering of oral prednisolone. (1D)
- We recommend adding lymphocyte-depleting Abs for acute TCMR that do not respond to corticosteroids, those above Banff Grade I, and for recurrent TCMRs (2 C).
- We recommend treating acute ABMR with IVIg and plasma exchange with or without anti-CD20, corticosteroids, or lymphocyte-depleting Abs (2 C) guided by biopsy findings and clinical response (NG).
- We strongly recommend checking for compliance to immunosuppression treatment in patients with acute rejection (1A) particularly in late rejection (NG).
- We recommend intensifying maintenance immunosuppression following an acute rejection (see Box 13).
- We recommend adding or restoring maintenance prednisone in patients not on steroids who have a rejection episode (2D).
- We recommend adding or restoring maintenance tacrolimus in patients not on CNIs who have a rejection episode (2D).

Initial treatment of acute rejection with pulse steroids remains a ‘gold standard’. While response is awaited, the result of graft biopsy should be available, thereby confirming the diagnosis, categorising the rejection as being cellular, Ab-mediated or mixed, Banff scoring, and excluding other causes of acute graft dysfunction. In the absence of adequate response to steroids in cell-mediated rejection, or if the Banff score is above Grade A, a T-cell depleting Ab should be used. If the biopsy shows an ABMR, PLEX or immunoadsorption should be used, to be followed by low-dose IVIg (100 mg/kg) or high-dose IVIg (2 g/kg) with or without steroids have become the standard of care. Anti-CD20 (rituximab) is a second-line despite mixed evidence of its added benefit.

Since acute rejection constitutes an additional immunological risk, it is imperative to review the patient’s compliance to immunosuppressive drugs, restore steroids and CNIs if withdrawn or minimised, and increase the target blood levels of CNIs. (See Box 12)

### BOX 15: Post-KT antimicrobial prophylaxis in KTRs

- We strongly recommend antimicrobial prophylaxis in all recipients (1A, 85.7% of survey respondents), to be individually tailored according to the recipient’s age, history of previous infection(s), comorbid conditions, status of specific immunity, and strength of immunosuppression (NG).

Chemoprophylaxis is a routine recommendation in most KT guidelines and experiences worldwide. It targets organisms that are often quiescent due to natural or vaccine-boosted immunity and may recrudesce with immunosuppression. The most commonly used agents are shown in Table 6. It is noteworthy that most responders to our survey include co-trimoxazole in their routine protocols. About 57% use CMV prophylaxis, most often in the D+/R – setting. All other recommended prophylactic agents are used by <20% of responders.

**Table 6.** Post-KT antimicrobial prophylaxis

Agent	Anti-microbial	Survey users, %	Indications	Recommendation	Duration	Grade
CMV	Acyclovir/valacyclovir	57.1	D+/R+	Recommended	3 months	2B
			D–/R+	Suggested		3B
	Valganciclovir		D+/R–	Strongly recommended	6 months	1A
	Acyclovir/valacyclovir		All KTRs: after T-cell depleting treatment	Strongly recommended	3 months	1 C
HSV	Acyclovir, valacyclovir, or famciclovir	10.7	Frequent recurrence	Recommended		2D
VZV	IVIg		<96 h following exposure	Recommended	Single dose	2D
	Oral acyclovir	10.7	For 7–10 days following exposure	Recommended	7 days	2D
HBV	Tenofovir, entecavir		HBs Ab-positive	Recommended	2 years or more until HBs Ab turns negative by 3-month check	2B
	Lamivudine (risk of resistance)			Suggested		3B
TB	Isoniazide		History of previous infection	Recommended	≥9 months	2D
UTI	Co-trimoxazole*	96.4	All recipients unless allergic	Recommended	≥6 months	2B
Pneumocystis jirovecii	Co-trimoxazole**		During and after treatment of acute rejection	Recommended	6 weeks	2 C
Candida	Fluconazole oral tablets	17.9	All recipients	Recommended	1 month	2 C
Oral Candida	Mycostatin			Suggested		NG

*Or a quinolone if allergic, **OR pentamidine if allergic.

### BOX 16: Post-KT monitoring of graft function

- We suggest monitoring graft function by measuring the urine volume, urinary protein excretion, serum creatinine (and eGFR), and performing US examination at a decremented frequency with progression of post-KT duration (Table 7).

**Table 7.** Suggested schedule for monitoring of graft function

	First 24 h	Hospital stay	Month 1	Months 2–3	Months 4–6	Annual	Recommendation
Urine volume (2 C)	Every 1–2 h	daily	As necessary for the management of complications				1D
Urinary protein:creatinine ratio (2 C)			@month 1	@month 3	@month 6	@month 6	2D
Serum creatinine (1B) eGFR (2D)	Twice	daily	2–3/week	weekly	/2 weeks	/1–3 months	2 C
Ultrasonography (2 C)	Once		Once		Once	Once	2D

### BOX 17: Post-KT pregnancy

- We recommend female KTRs attempting pregnancy to be at least 1 year post-KT (2 C) AND fulfilling the following:

- Stable kidney function with serum creatinine not exceeding 1.5 mg/dL without rejection episodes in the past year (2 C).
- Proteinuria not exceeding 1 g/day (2 C).
- Well controlled blood pressure.

- No active CMV infection or at least 6 months following last active CMV infection.
- We strongly recommend modifying the immunosuppression protocol in women in the setting of attempted or realised pregnancy as mentioned in Box 13

### BOX 18: Protocol/surveillance biopsy

- We strongly recommend a graft biopsy in the setting of persisting delayed graft function (1 C).
- We strongly recommend a graft biopsy following treatment of acute rejection episodes if serum creatinine does not return to baseline (1 C).
- We suggest that all very-high- and high-risk KTRs undergo a scheduled graft biopsy (with staining for C4d) at 3–6 months post-KT and 1–3 times thereafter for the first 2 years (NG).
- We suggest at least one graft biopsy (with staining for C4d) in intermediate-risk KTRs during the first year [59] (NG).
- We suggest that any KTR with detected *de novo* DSA, or with one biopsy confirmed ABMR episode or evidence of non-adherence to undergo at least one protocol biopsy (NG).
- We suggest that a protocol biopsy be obtained prior to a shift in the maintenance immunosuppression protocol and at 3 months thereafter (NG).
- We suggest that a protocol biopsy to be obtained in KTRs with a high risk of recurrence of their original disease (NG).

The objective of a surveillance biopsy is to detect subclinical rejection, certain viral infections, drug toxicity or recurrence/*de novo* glomerulonephritis (GN). The potential benefit of early treatment is opposed by potential risk of injury to a healthy graft. For this reason, individual centres diverge in including surveillance biopsy in their routine protocols. In our survey, 48% of responders favour protocol biopsies. Evidence continues to show a negative impact on graft survival in patients with subclinical TCMR and ABMR, thereby tipping the balance in favour of routine protocol biopsy at least until non-invasive rejection biomarkers have been developed, as predicted by 16% of our survey respondents.

### BOX 19: Monitoring for donor-specific Abs (DSAs)

- We recommend that a serum sample should be sent at the time of renal biopsy (for acute or chronic graft dysfunction) to look for HLA-specific antibodies (2 C).
- We suggest that monitoring of post-KT DSA be performed in very high- and high-risk patients during the first 2 years post-KT [60] (NG).
- We suggest screening for anti-HLA Abs in intermediate- and low-risk KTRs at 3 months post-KT, then once annually for the first 2 years post-KT with a concurrent biopsy if positive [61] (NG).
- We suggest screening for anti-HLA Abs in recipients under minimisation protocols every 3 months for 1 year, and once annually for the first 2 years post-KT (NG).

Acute ABMR can occur at any time after KT due to the development of *de novo* DSA (dnDSA). Recent literature reports ~20% incidence of dnDSA in immunological low- or moderate-risk patients. This occurs within the first year after KT with a range of 3–24 months. The incidence increases with non-adherence to immunosuppressive medication. dnDSA could also be associated with both clinical and subclinical TCMR.

It is recommended to stain all biopsies for C4d and to send a serum sample for detection of HLA-specific Abs, in order to facilitate the diagnosis of ABMR in the setting of C4d negativity. This is consistent with joint British Society for Histocompatibility and Immunogenetics (BSHI)/BTS guidelines and those of the Transplantation Society. It is also in agreement with the local survey with a 56% positive response for DSA screening.

### BOX 20: Acute rejection

- We recommend considering the diagnosis of acute rejection, as well as other causes of decline of graft function, in KTRs who meet the criteria of acute kidney injury (NG).
- We strongly recommend graft biopsy to confirm the clinical diagnosis of acute rejection before initiating its treatment, unless the biopsy is contraindicated or will substantially delay treatment (1 C).
- We recommend that two cores of renal tissue should be obtained at KT biopsy to increase the sensitivity of the investigation (2 C).
- We recommend early testing for serum anti-HLA Abs in patients with acute rejection (2B).
- We suggest testing for non-HLA Abs in patients with biopsy evidence of microvascular inflammation without C4d deposits and negative serum DSAs (NG).

Emphasis is made in most recent guidelines to confirm the diagnosis of acute rejection by biopsy (85.7% of survey respondents concur) and to simultaneously test for serum anti-HLA Abs (see guideline 9.9). Of the survey respondents, 21.4% required two or more of the following criteria to diagnose acute rejection: Clinical symptoms and signs (46.4%), rise of serum creatinine (75%), imaging (25%), and biomarkers (7.1%).

### BOX 21: Chronic graft dysfunction

- We suggest standard clinical, laboratory and imaging evaluation to categorise the cause of chronic graft dysfunction function (NG).
- We suggest graft biopsy in KTRs with non-obstructive progressively declining graft function (NG).
- We recommend testing for anti-HLA Abs in KTRs with histological evidence of chronic active rejection (2 C).
- We suggest chronic kidney disease (CKD) staging in patients with chronic graft dysfunction and implementing standard management protocols accordingly (NG).

Progressive non-obstructive decline of graft function may be attributed to chronic rejection, immunosuppressive drug toxicity, recurrent/*de novo* GN, infection, graft arterial stenosis or other kidney disease. Graft biopsy is mandatory to establish the diagnosis. Traditionally, little can be done to help those with chronic rejection. When chronic ABMR is recognised, histologically and/or serologically, as a common cause of chronic rejection, more targeted treatment protocols, including steroids anti-CD20 or even anti-CD19 and other biological agents have been applied with variable responses, albeit being so far generally unfavourable.

### BOX 22: Monitoring for recurrence of original disease/de novo GN

- We suggest considering recurrence of the original disease or *de novo* GN in KTRs who develop proteinuria or other urinary abnormalities and/or progressively deteriorating graft function. (NG)
- We suggest performing non-invasive laboratory tests (e.g. anti-anti-phospholipase A2 receptor [PLA2R], anti-GBM, anti-neutrophilic cytoplasmic Ab [ANCA], etc.) for diagnosis and follow-up of recurrent disease or *de novo* GN (NG).
- We suggest obtaining a graft biopsy when recurrence or *de novo* GN is suspected. (NG) OR if sustained new onset proteinuria develops (protein:creatinine ratio >50 mg/mmol or albumin:creatinine ratio >35 mg/mmol) (2 C).

Many kidney diseases may recur in the graft, particularly those of immunological nature. The chances and average timing of recurrence of different diseases are fairly well known, hence the feasibility of early detection even by routine follow-up and surveillance biopsy. The same applies to *de novo* GN.

### BOX 23: Monitoring drug levels

- We suggest routine checking for the potential adverse reactions of the immunosuppressive drugs (NG).
- We suggest, whenever applicable, to measure the blood level of drugs known to interact with CNIs for initial dose adjustment and with changes in CNI doses or blood levels (NG).

It is imperative to monitor KTRs for clinical side-effects, blood counts, liver and kidney functions for adverse drug reactions. Blood levels of CNIs must be monitored as explained in Box 12. The mTORi blood levels are also mandatory, and MMF levels preferable. Therapeutic blood level monitoring extends beyond the immunosuppressive drugs to many others that utilise the cytochrome P450/P-glycoprotein system for their absorption and metabolism. If measurement of blood levels is not feasible, careful clinical observation for adverse reactions is mandatory.

### BOX 24: Monitoring for post-KT infection

*Known previous chronic infection*

- We suggest assessment of KTRs for re-activation of pre-existing chronic infection at 3 months post-KT and as frequently as necessary thereafter (NG).

*Cytomegalovirus (CMV)*

- In those who had received induction therapy, we suggest screening all KTRs (except if recipient negative/donor negative [R–/D–]) for active CMV infection every 3 months (1A–C) by detecting serum CMV-specific IgM, four-fold rise in IgG, pp65 rapid antigen (NG).

- For 1 year if R+/D+
- For 2 years if R–/D+

- We suggest repeat screening for CMV infection 3 months after stopping universal prophylaxis [62] (NG).

*BK Polyomavirus (BKV)*

- We recommend screening all KTRs for BKV viral load or by performing urine microscopy for decoy cells or by PCR on urine or serum (2 C) monthly for the first 9 months (2D) then every 3 months until 2 years post-KT (2B), whenever there is an unexplained rise in serum creatinine and after treatment for acute rejection (2D).
- We recommend that renal biopsies with SV40 staining should be done in patients with suspected polyomavirus nephropathy or in chronically deteriorating graft function (2 C).
- We recommend reduction of immunosuppressive medications in patients with BKV viral load of >1000 copies/mL sustained for 3 weeks or >10 000 copies/mL (2 C).

*Epstein–Barr virus (EBV)*

- We recommend monitoring high-risk KTRs (R–/D+) for EBV by nucleic acid test (2 C) once in the first week, then monthly for the first 3–6 months and every 3 months until the end of the first post-KT year (2D).
- We recommend monitoring high-risk KTRs for EBV following treatment of acute rejection (2D).

*Hepatitis B virus (HBV)*

- We suggest measuring the serum level of HB Ab titre in HB-negative or -positive KTRs and maintaining a level >10 IU/mL by vaccination, or administration of booster doses [17].

The list of bacterial, viral, fungal and parasitic [63] diseases that can affect KTRs is beyond the scope of this set of guidelines. Emphasis is made only on the mentioned four viral infections, as they are traceable by specific biomarkers in the absence of any symptoms. Unfortunately, only 24% of our survey respondents include monitoring for viral markers in their routine protocols.

### BOX 25: Post-KT diabetes mellitus

- We strongly recommend screening all non-diabetic KTRs with fasting plasma glucose, oral glucose tolerance testing, and/or HbA1c (1 C) at least:

- Weekly for 4 weeks (2D);
- Every 3 months for 1 year (2D); and
- Annually, thereafter (2D).

- We recommend screening for new-onset diabetes after KT with fasting glucose, oral glucose tolerance testing, and/or HbA1c after starting, or substantially increasing the dose, of CNIs, mTORi, or corticosteroids (2D).

Pre-transplant diabetes is often ameliorated in CKD due to impaired clearance of insulin and many glucose lowering agents, despite the peripheral tissue insulin resistance. This perturbation is corrected as kidney function is restored by a successful KT. Unfortunately, corticosteroids, CNIs (particularly tacrolimus), and mTORis are diabetogenic, thereby leaching the benefit of restored kidney function, and prompting the development of new onset diabetes in non-diabetic KTRs.

### BOX 26: Cardiovascular disease

- We strongly recommend measuring blood pressure at each clinic visit (1 C).
- We suggest assessment for cardiovascular disease in all KTRs before, 3 months after KT and then annually (NG).
- We suggest assessment for obesity in all KTRs before, 3 months after, and 6-monthly thereafter including (NG):

- Measure height and weight and calculating BMI at each visit, at each visit.
- Measurement of a complete lipid profile in all adult and adolescent KTRs.
- Measurement of a complete lipid profile 2–3 months after a change in treatment or other conditions known to cause dyslipidaemias

Cardiovascular events occur in 3.5–5% of KTRs every year, reflecting a significantly high risk compared to the general population. This is attributed to a large number of ‘traditional’ and ‘non-traditional’ risk factors that tend to persist after KT. The latter adds a further drug-related risk, attributed to corticosteroids and CNIs. Therefore, it is mandatory to include event and risk factor monitoring of KTRs.

### Box 27: Post-KT bone disease

- In patients in the immediate post-KT period, we strongly recommend measuring serum calcium and phosphate at least weekly, until stable (1B) then every 3–6 months for as long as necessary depending on the stage of post-KT CKD stage, patient’s age, and the extent of metabolic bone disease (MBD).
- We suggest measuring serum vitamin D (2 C) and parathormone levels (NG), every 3–6 months for as long as necessary depending on the stage of post-KT CKD stage, patient’s age, and the extent of MBD.
- We suggest measuring bone mineral density by dual-energy X-ray absorptiometry (DXA) or CT scanning at 3 months post-KT to estimate fracture risk (NG).
- We suggest post-KT measurement of bone mineral density every 6 months for high-fracture risk KTRs and annually for low-fracture risk patients (NG).
- We recommend avoiding, or using the lowest therapeutic doses, of immunosuppressive drugs that have a negative impact on bone health (NG).

CKD-associated bone disease continues after KT and often worsens despite normal graft function, owing to the use of corticosteroids. CNIs may increase serum parathormone levels. Sirolimus is incriminated in deterioration of post-KT osteodystrophy. On the other hand, combination of everolimus and MMF in a steroid-free, CNI-free protocol seems to be bone-protective [64,65]^66^].

Post-KT osteodystrophy is often associated with mineral abnormalities, hypovitaminosis D, and persistent hyperparathyroidism, hence the mentioned monitoring recommendations. In addition, osteonecrosis may develop *de novo*, which is mainly attributed to the use of corticosteroids. The role of other immunosuppressive drugs in this complication is uncertain.

### BOX 28: Post-KT malignancy

- We recommend pre-KT and at least one annual post-KT tumour screening by clinical examination (including dermatological and lymph nodal), fecal blood and tumour markers as per local guidelines.
- We recommend chest imaging prior to KT in all KTRs (2 C).
- We recommend chest CT for current or former tobacco users with a > 30 pack-year history, as per local guidelines, and chest radiograph for other KTRs (2 C).
- We strongly recommend screening for RCC with US for KTRs at increased risk, such as a long time on dialysis, family history of renal cancer, acquired cystic disease, and analgesic nephropathy (1D).
- We strongly recommend screening for bladder carcinoma using urine cytology or cystoscopy for KTRs at increased risk, such as schistosomiasis, previous cyclophosphamide use or history of heavy smoking (>30 pack-year) (1D).
- We strongly recommend screening for hepatocellular carcinoma in KTRs with cirrhosis prior to KT using techniques (e.g., US, α-fetoprotein, etc.) (1 C).
- We strongly recommend screening for bowel cancer in KTRs with inflammatory bowel disease as per local guidelines (1 C).
- We recommend screening for cervical cancer (PAP smear), for female recipients annually after KT (1 C).
- We recommend breast cancer screening in all female recipients aged 50–69 years every 1–2 years (1 C).

The incidence of post-KT myeloproliferative disease is multiplied many folds post-KT. This is attributed to immunosuppression (e.g. CNIs, MMF), the use of T-cell depleting agents and to certain viral infections (e.g. EBV, CMV).

The occurrence of other cancers is also significantly increased in KTRs, with increasing incidence with advancing age >65 years. The most notable are non-melanoma skin cancers and bronchial, urinary, hepatic and bowel malignancy.

Obviously, early detection of these cancers is mandatory for adequate specific treatment and modification of immunosuppressive therapy. The latter often includes switching to mTORis [66]. Unfortunately, only 20% of our survey’s respondent included routine monitoring for tumour markers.

### Box 29: Annual review

- We suggest that every transplant centre establishes a multispecialty annual review clinic for the appraisal of its KTRs at their KT anniversaries

### BOX 30: Special populations

The high success rate of KT has alleviated a lot of concerns in children and the elderly. Many female KTRs can complete successful pregnancies and deliver normal babies while on immunosuppressive therapy. However, there are issues that must be taken into consideration regarding vaccination, risk assessment, induction, choice of immunosuppressive drugs and their doses, and subsequent monitoring.

Table 8 shows the perception of our survey respondents regarding the modification of immunosuppression protocols in the mentioned special populations.

**Table 8.** Survey respondents recommended modifications in special populations

	% of respondents recommending modification		
	Post-KT pregnancy	Children	Aged >60 years
Vaccination	29.6	50.0	40.0
Risk assessment	48.2	53.6	48.0
Desensitisation		14.3	4.0
Induction		57.1	28.0
Maintenance	62.9	67.9	72.0
Chemoprophylaxis	14.8	39.2	44.0
Monitoring	44.4	39.3	32.0

We have alluded to some recommendations concerning these populations in the various sections of this Guideline. However, it is strongly recommended to collaborate with respective specialists and refer to specific guidelines for the adequate management of individual cases.

## References

[1] Kidney Disease: Improving Global Outcomes (KDIGO) Transplant Work Group. KDIGO clinical practice guideline for the care of kidney transplantrecipients. Am J Transplant. 2009;9(Suppl. 3): S1–S155. 10.1111/j.1600-6143.2009.02834.x 19845597

[2] Kidney Disease Improving Global Outcomes (KDIGO). Living Kidney Donor Guidelines, 2017. cited 12 2020 Available at: https://kdigo.org/guidelines/living-kidney-donor/

[3] Kidney Disease Improving Global Outcomes (KDIGO). Transplant Candidate Guidelines, 2020. cited 12 2020. Available at: https://kdigo.org/guidelines/transplant-candidate/

[4] European Association of Urology (EAU). EAU Guidelines on Renal Transplantation. cited 12 2020. Available at: https://uroweb.org/wp-content/uploads/EAU-Guidelines-on-Renal-Transplantation-2018-large-text.pdf

[5] British Transplantation Society (BTS). Guidelines for antibody incompatible transplantation, Third. Available at: https://bts.org.uk/wp-content/uploads/2016/09/02_BTS_Antibody_Guidelines-1.pdf.

[6] Jones-Hughes T, Snowsill T, Haasova M, et al. Immunosuppressive therapy for kidney transplantation in adults: a systematic review and economic model. Health Technol Assess. 2016;20:1–594. 10.3310/hta20620 PMC5018688 27578428

[7] Canadian Clinical Guidelines. Clinical guidelines for kidney transplantation. cited 12 2020. Available at: http://www.transplant.bc.ca/documents/health%20professionals/clinical%20guidelines/clinical%20guidelines%20for%20kidney%20transplantation.pdf

[8] Heemann U, Abramowicz D, Spasovski G, et al. Endorsement of the kidney disease improving global outcomes (KDIGO) guidelines on kidney transplantation: a European renal best practice (ERBP) position statement. Nephrol Dial Transplant. 2011;26(7):2099–2106. 21555392 10.1093/ndt/gfr169

[9] Renal Unit at the Royal Infirmary of Edinburgh and the University of Edinburgh. Edren Renal Transplantation Handbook, 2019. cited 12 2020. Available at: https://edren.org/ren/handbook/transplant-handbook/

[10] Government of Canada. Immunization of immunocompromised persons: canadian immunization guide. cited 12 2020. Available at: https://www.canada.ca/en/public-health/services/publications/healthy-living/canadian-immunization-guide-part-3-vaccination-specific-populations/page-8-immunization-immunocompromised-persons.html

[11] Egyptian Society of Pediatric Nephrology and Transplantation. ESPNT protocols in pediatric kidney transplantation. cited 12 2020. Available at: http://cairopedneph.com/document/Tx%20guidelines6.pdf

[12] Segall L, Nistor I, Pascual J, et al. Criteria for and appropriateness of renal transplantation in elderly patients with end-stage renal disease: a literature review and position statement on behalf of the European renal association-european dialysis and transplant association descartes working group and European renal best practice. Transplantation. 2016;100(10):e55–65. 27472096 10.1097/TP.0000000000001367

[13] Cabiddu G, Spotti D, Gernone G, et al. A best-practice position statement on pregnancy after kidney transplantation: focusing on the unsolved questions. The kidney and pregnancy study group of the Italian society of nephrology. J Nephrol. 2018;31:665–681. 29949013 10.1007/s40620-018-0499-x PMC6182355

[14] Danziger-Isakov L, Kumar D, ID AST. Community of practice. Vaccination of solid organ transplant candidates and recipients. Clin Transplant. 2019;33(9):e13563. 31002409 10.1111/ctr.13563

[15] Tornesello ML, Loquercio G, Tagliamonte M, et al. Human papillomavirus infection in urine samples from male renal transplant patients. J Med Virol. 2010;82(7):1179–1185. 20513081 10.1002/jmv.21784

[16] Katerinis I, Hadaya K, Duquesnoy R, et al. De novo anti‐HLA antibody after pandemic H1N1 and seasonal influenza immunization in kidney transplant recipients. Am J Transplant. 2011;11(8):1727–1733. 21672157 10.1111/j.1600-6143.2011.03604.x

[17] Chong PP, Avery RK. A comprehensive review of immunization practices in solid organ transplant and hematopoietic stem cell transplant recipients. Clin Ther. 2017;39(8):1581–1598. 28751095 10.1016/j.clinthera.2017.07.005

[18] Keith DS, Vranic GM. Approach to the highly sensitized kidney transplant candidate. Clin J Am Soc Nephrol. 2016;11(4):684–693. 26915916 10.2215/CJN.05930615 PMC4822662

[19] Severova G, Nikolov I, Sikole A, et al. Clinical importance of non-donor-specific HLA antibodies and possible impact on graft histology in kidney transplant recipients – 12 months protocol biopsy study. Transplantation. 2018;102:S484.

[20] Bakr MA, Shehab El-Dein AB, Refaie AF, et al. Renal transplantation in Mansoura, Egypt. Transplantation. 2020;104(8):1519–1521. 32732819 10.1097/TP.0000000000003268

[21] Shokeir AA. Open versus laparoscopic live donor nephrectomy: a focus on the safety of donors and the need for a donor registry. J Urol. 2007;178(5):1860–1866. 17868736 10.1016/j.juro.2007.07.008

[22] Mansour AM, El-Nahas AR, Ali-El-Dein B, et al. Enhanced recovery open vs laparoscopic left donor nephrectomy: a randomized controlled trial. Urology. 2017;110:98–103. 28893633 10.1016/j.urology.2017.08.047

[23] El-Agroudy AE, Sabry AA, Wafa EW, et al. Long-term follow-up of living kidney donors: a longitudinal study. BJU Int. 2007;100(6):1351–1355. 17941927 10.1111/j.1464-410X.2007.07054.x

[24] Wagenaar S, Nederhoed JH, Hoksbergen AW, et al. Minimally invasive, laparoscopic, and robotic-assisted techniques versus open techniques for kidney transplant recipients: a systematic review. Eur Urol. 2017;72(2):205–217. 28262412 10.1016/j.eururo.2017.02.020

[25] Kaminska D, Koscielska-Kasprzak K, Chudoba P, et al. The influence of warm ischemia elimination on kidney injury during transplantation – clinical and molecular study. Sci Rep. 2016;6(1):36118. 27808277 10.1038/srep36118 PMC5093711

[26] Wang K, Zhang P, Xu X, et al. Right versus left laparoscopic living-donor nephrectomy: A meta-analysis. Exp Clin Transplant. 2015;13(3):214–226. 26086831

[27] El-Sherbiny M, Abou-Elela A, Morsy A, et al. The use of inferior epigastric artery for accessory lower polar artery revascularization in live donor renal transplantation. Int Urol Nephrol. 2008;40(2):283–287. 17721826 10.1007/s11255-007-9257-z

[28] Osman Y, Ali-El-Dein B, Shokeir A, et al. Routine insertion of ureteral stent in live-donor renal transplantation: is it worthwhile? Urology. 2005;65(5):867–871. 15882713 10.1016/j.urology.2004.11.050

[29] Soliman SA, Shokeir AA, El-Hefnawy AS, et al. Vascular and haemorrhagic complications of adult and paediatric live-donor renal transplantation: A single-center study with long-term follow-up. Arab J Urol. 2012;10(2):155–161. 26558019 10.1016/j.aju.2011.12.002 PMC4442900

[30] Dimitroulis D, Bokos J, Zavos G, et al. Vascular complications in renal transplantation: a single-center experience in 1367 renal transplantations and review of the literature. Transplant Proc. 2009;41(5):1609–1614. 19545690 10.1016/j.transproceed.2009.02.077

[31] Osman Y, Shokeir A, Ali-el-Dein, et al. Vascular complications after live donor renal transplantation: study of risk factors and effects on graft and patient survival. J Urol. 2003;169(3):859–862. 12576799 10.1097/01.ju.0000050225.74647.5a

[32] Shokeir AA, Osman Y, Ali-El-Dein B, et al. Surgical complications in live-donor pediatric and adolescent renal transplantation: study of risk factors. Pediatr Transplant. 2005;9(1):33–38. 15667608 10.1111/j.1399-3046.2005.00244.x

[33] Choate HR, Mihalko LA, Choate BT. Urologic complications in renal transplantation. Transl Androl Urol. 2019;8(2):141–147. 31080774 10.21037/tau.2018.11.13 PMC6503228

[34] Giustacchini P, Pisanti F, Citterio F, et al. Renal vein thrombosis after renal transplantation: an important cause of graft loss. Transplant Proc. 2002;34(6):2126–2127. 12270338 10.1016/s0041-1345(02)02876-2

[35] Granata A, Clementi S, Londrino F, et al. Renal transplant vascular complications: the role of Doppler ultrasound. J Ultrasound. 2014;18(2):101–107. 26191097 10.1007/s40477-014-0085-6 PMC4504861

[36] Ghazanfar A, Tavakoli A, Augustine T, et al. Management of transplant renal artery stenosis and its impact on long-term allograft survival: a single-centre experience. Nephrol Dial Transplant. 2011;26(1):336–343. 20601365 10.1093/ndt/gfq393

[37] Osman Y, Kamal M, Soliman S, et al. Necessity of routine postoperative heparinization in non-risky live-donor renal transplantation: results of a prospective randomized trial. Urology. 2007;69(4):647–651. 17445644 10.1016/j.urology.2006.12.017

[38] Harraz AM, Shokeir AA, Soliman SA, et al. Salvage of grafts with vascular thrombosis during live donor renal allotransplantation: a critical analysis of successful outcome. Int J Urol. 2014;21(10):999–1004. 24861882 10.1111/iju.12485

[39] Salehipour M, Salahi H, Jalaeian H, et al. Vascular complications following 1500 consecutive living and cadaveric donor renal transplantations: a single center study. Saudi J Kidney Dis Transpl. 2009;20(4):570–572. 19587495

[40] Elmekresh M, Osman Y, Ali-El-Dein B, et al. Urological complications after living‐donor renal transplantation. BJU Int. 2001;87(4):295–306. 11251519

[41] Kayler L, Kang D, Molmenti E, et al. Kidney transplant ureteroneocystostomy techniques and complications: review of the literature. Transplant Proc. 2010;42(5):1413–1420. 20620446 10.1016/j.transproceed.2010.04.016

[42] Abrol N, Dean PG, Prieto M, et al. Routine stenting of extravesical ureteroneocystostomy in kidney transplantation: a systematic review and meta-analysis. Transplant Proc. 2018;50(10):3397–3404. 30577212 10.1016/j.transproceed.2018.06.041

[43] Saad IR, Habib E, ElSheemy MS, et al. Outcomes of living donor renal transplantation in children with lower urinary tract dysfunction: a comparative retrospective study. BJU Int. 2016;118(2):320–326. 26434410 10.1111/bju.13347

[44] Gdor Y, Gabr A, Faerber G, et al. Holmium:yttrium-aluminum-garnet laser endoureterotomy for the treatment of transplant kidney ureteral strictures. Transplantation. 2008;85(9):1318–1321. 18475190 10.1097/TP.0b013e31816c7f19

[45] Ulrich F, Niedzwiecki S, Fikatas P, et al. Symptomatic lymphoceles after kidney transplantation - multivariate analysis of risk factors and outcome after laparoscopic fenestration. Clin Transplant. 2010;24(2):273–280. 19719727 10.1111/j.1399-0012.2009.01073.x

[46] Lucewicz A, Wong G, Lam V, et al. Management of primary symptomatic lymphocele after kidney transplantation: a systematic review. Transplantation. 2011;92(6):663–673. 21849931 10.1097/TP.0b013e31822a40ef

[47] Helfand BT, Newman JP, Mongiu AK, et al. Reconstruction of late-onset transplant ureteral stricture disease. BJU Int. 2011;107(6):982–987. 20825404 10.1111/j.1464-410X.2010.09559.x

[48] Cheungpasitporn W, Thongprayoon C, Mao MA, et al. Incidence of kidney stones in kidney transplant recipients: A systematic review and meta-analysis. World J Transplant. 2016;6(4):790–797. 28058231 10.5500/wjt.v6.i4.790 PMC5175239

[49] Oliveira M, Branco F, Martins L, et al. Percutaneous nephrolithotomy in renal transplants: a safe approach with a high stone-free rate. Int Urol Nephrol. 2011;43(2):329–335. 20848196 10.1007/s11255-010-9837-1

[50] Stravodimos KG, Adamis S, Tyritzis S, et al. Renal transplant lithiasis: analysis of our series and review of the literature. J Endourol. 2012;26(1):38–44. 22050494 10.1089/end.2011.0049

[51] Molenaar NM, Minnee RC, Bemelman FJ, et al. Vesicoureteral reflux in kidney transplantation. Prog Transplant. 2017;27(2):196–199. 28617157 10.1177/1526924817699965

[52] Pichler R, Buttazzoni A, Rehder P, et al. Endoscopic application of dextranomer/hyaluronic acid copolymer in the treatment of vesico-ureteric reflux after renal transplantation. BJU Int. 2011;107(12):1967–1972. 21059169 10.1111/j.1464-410X.2010.09792.x

[53] Nie Z, Zhang K, Huo W, et al. Comparison of urological complications with primary ureteroureterostomy versus conventional ureteroneocystostomy. Clin Transplant. 2010;24(5):615–619. 19925475 10.1111/j.1399-0012.2009.01134.x

[54] El-Dahshan KF, Bakr MA, Donia AF, et al. Co-administration of ketoconazole to tacrolimus-treated kidney transplant recipients: a prospective randomized study. Nephrol Dial Transplant. 2004;19(6):1613–1617. 15034161 10.1093/ndt/gfh191

[55] Banerjee A, Julie BM, Sharma A, et al. Steroid withdrawal protocols in renal transplantation. Arch Clin Nephrol. 2018;4:001–008.

[56] Tedesco-Silva H, Pascual J, Viklicky O, et al. Safety of everolimus with reduced calcineurin inhibitor exposure in de novo kidney transplants: an analysis from the randomized TRANSFORM study. Transplantation. 2019;103(9):1953–1963. 30801548 10.1097/TP.0000000000002626

[57] Gheith OA, Nematalla AH, Bakr MA, et al. Steroid avoidance reduce the cost of morbidities after live-donor renal allotransplants: a prospective, randomized, controlled study. Exp Clin Transplant. 2011;9(2):121–127. 21453230

[58] Barsoum RS, Morsy AA, Iskander IR, et al. The Cairo kidney center protocol for rapamycin-based sequential immunosuppression in kidney transplant recipients: 2-year outcomes. Exp Clin Transplant. 2007;5(2):649–657. 18194116

[59] Cherukuri A, Mehta R, Sharma A, et al. Post-transplant donor specific antibody is associated with poor kidney transplant outcomes only when combined with both T-cell–mediated rejection and non-adherence. Kidney Int. 2019;96(1):202–213. 31029504 10.1016/j.kint.2019.01.033

[60] Süsal C, Aykut G, Morath C, et al. Relevance of donor‐specific antibody monitoring after kidney transplantation: findings from the collaborative transplant study and the heidelberg transplant center. HLA. 2019;94(Suppl. S2):11–15. 10.1111/tan.13665 31403240

[61] Viglietti D, Loupy A, Vernerey D, et al. Value of donor-specific anti-HLA antibody monitoring and characterization for risk stratification of kidney allograft loss. J Am Soc Nephrol. 2017;28(2):702–715. 27493255 10.1681/ASN.2016030368 PMC5280026

[62] Singh N. Preemptive therapy versus universal prophylaxis with ganciclovir for cytomegalovirus in solid organ transplant recipients. Clin Infect Dis. 2001;32(5):742–751. 11229841 10.1086/319225

[63] Barsoum RS. Parasitic infections in transplant recipients. Nat Clin Pract Nephrol. 2006;2(9):490–503. 16941042 10.1038/ncpneph0255

[64] Hadji P, Coleman R, Gnant M. Bone effects of mammalian target of rapamycin (mTOR) inhibition with everlolimus. Crit Rev Oncol Hematol. 2013;87:101–111. 23838481 10.1016/j.critrevonc.2013.05.015

[65] Chen J, Long F. mTOR signaling in skeletal development and disease. Bone Res. 2018;6:1. 29423330 10.1038/s41413-017-0004-5 PMC5802487

[66] Knoll GA, Kokolo MB, Mallick R, et al. Effect of sirolimus on malignancy and survival after kidney transplantation: systematic review and meta-analysis of individual patient data [published correction appears in BMJ. BMJ. 2014;349(nov24 1):g6679. 25422259 10.1136/bmj.g6679 PMC4241732

